# IC261, a specific inhibitor of CK1δ/ε, promotes aerobic glycolysis through p53-dependent mechanisms in colon cancer

**DOI:** 10.7150/ijbs.40960

**Published:** 2020-01-17

**Authors:** Min Liu, Yuhan Hu, Shuya Lu, Manman Lu, Jingsong Li, Haimin Chang, Huijie Jia, Min Zhou, Feng Ren, Jiateng Zhong

**Affiliations:** 1 Department of Pathology, Xinxiang Medical University, Henan, China; 2 Department of Pathology, The First Affiliated Hospital of Xinxiang Medical University, Henan, China; 3 The Academic Affairs Office, Xinxiang Medical University, Henan, China

**Keywords:** colon cancer, casein kinase, aerobic glycolysis, IC261

## Abstract

Casein kinase 1δ (CK1δ) and casein kinase 1ε (CK1ε) have been proposed to be involved in DNA replication, differentiation and apoptosis, thus participating in the regulation of tumorigenesis. However, their functions in colon cancer and the underlying mechanism remain unclear. Here, we found that the expression of CK1ε and CK1δ increased significantly in cancer tissues and the upregulation of CK1ε and CK1δ were closely related to poor differentiation, advanced TNM stage and poor prognosis of colon cancer. CK1δ/ε inhibitor IC261 could induce a decrease in cell survival and proliferation, and an increase in apoptosis in colon cancer cells. Interestingly, IC261 increased the level of aerobic glycolysis in colon cancer cells. Meanwhile, IC261 caused the decrease of p53 protein level and the misregulation of glycolysis related genes (TIGAR, G6PD, GLUT1) which are closely related to the regulation of glycolysis by p53. Inhibiting p53 by siRNA or inhibitor could significantly attenuate the upregulation of aerobic glycolysis induced by IC261. Finally, inhibition of aerobic glycolysis can further increase the cytotoxicity induced by IC261. Collectively, our results revealed that IC261 could inhibit the growth of colon cancer cells and increase the level of aerobic glycolysis, which is regulated by p53-dependent manner. This result suggested that targeting CK1δ/ε and glycolysis might be a valuable strategy treatment and combination therapies for colon cancer.

## Introduction

In combination of both sexes, the colorectal cancer (CRC) ranks third in incidence and second in mortality worldwide 1. CRC accounts for about one-tenth of all cancers, which seriously affects human health 2. Although traditional treatment can improve the life span of patients, patients with CRC still show a high recurrence rate and mortality 3. Over the past decade, with the advancement of precision medicine and the development of targeted drugs, more and more molecular targets have been found and studied in CRC 4.

Casein kinase 1 (CK1) is a family of ubiquitous serine/threonine protein kinases containing seven family members α, β, γ1, γ2, γ3, δ and ε 5. Numerous studies have reported that CK1 kinase can regulate a variety of cellular biological behaviors, including DNA replication, differentiation, proliferation, membrane trafficking signal transduction and apoptosis 6-9.Based on these biological regulatory effects, it is not surprising that alterations and mutations in the expression of CK1 are involved in carcinogenesis 10, 11. Some small molecule inhibitors have been synthesized to clarify the mechanism of CK1 and develop clinical targeted drugs 12. Among these inhibitors, IC261 has been the most widely studied, which targets CK1δ and CK1ε 13, 14. Few literatures have reported the relationship between CK1 family and colorectal cancer, however, the investigations only limited to the expression of CK1 in clinical tissue samples. The effect of IC261 on the biological behavior of colon cancer cells and the underlying mechanism are still unclear 15.

Glucose metabolic alteration is a hallmark of tumor cells. Unlike normal cells which obtain energy through mitochondrial respiration, tumor cells prefer to use glycolysis to obtain energy, even when oxygen is sufficient, known as Warburg effect or aerobic glycolysis 16, 17.Limited ability of glycolysis to obtain ATP promotes increased glucose uptake and utilization in tumor cells 18. However, aerobic glycolysis of cancer cells can not only provide precursors for cell synthesis of biomacromolecules, but also produce a large number of antioxidants 19.Recent studies have shown that aerobic glycolysis of cancer cells plays an important role in malignant progression 20-22. Targeting aerobic glycolysis has become a new direction of cancer control and treatment. Although many cancer-related genes are involved in the regulation of aerobic glycolysis, it is unknown whether CK1 is involved in this process.

In this research, we systematically explored the relationship between CK1δ/ε expression and clinical characteristics in colon cancer, and clarified the effect of CK1δ/ε inhibitors IC261 on biological behavior of colon cancer cells. Meanwhile, we examined the effects of IC261 on aerobic glycolysis of colon cancer cells and explored their possible mechanisms. To some extent, our study may provide a theoretical basis for CK1 inhibitor in the treatment of colon cancer and supply a strategy of combination therapy.

## Materials and Methods

### Tissue specimens

Fresh colon cancer tissues and matched adjacent normal tissues were obtained from patients at the Department of General Surgery, the First Affiliated Hospital of Xinxiang Medical University (Xinxiang City, Henan Province, China). Formalin- fixed paraffin-embedded colon cancer colon cancer samples were obtained from the Department of Pathology, the First Affiliated Hospital of Xinxiang Medical University. All patients did not receive radiotherapy, chemotherapy, targeted therapy and immunotherapy before surgery. All patients were diagnosed with colon cancer by HE (Hematoxylin-Eosin) staining. The research was approved by the Medical Ethics Committees of Xinxiang Medical University.

### Quantitative Real-time PCR (qRT-PCR)

The total RNAs from tissues and cultured cells were extracted with Trizol reagent (Invitrogen, USA) according to the manufacturer's protocol. Then, the cDNAs were synthesized through reverse transcription (RT). The relative expression of CK1δ, CK1ε, GLUT1 (glucose transporter 1) were detected by Real Time PCR System (ABI7500, USA). The primer sequences were as follows: CK1δ (F:5'-GGATCCATGGAGCTGAGAGTCGGGAACAG-3', R:5'-GGATCCTCACGGTGCACGACAGACTGA-3'), CK1ε (F: 5'-ATGGAGTTGCGTGTTGGA-3', R: 5'-GTCAACATACAACACTTTCTG-3'), GLUT1 (F: 5'-ACCATTGGCTCCGGTATCG-3', R: 5'-GCTCGCTCCACCACAAACA-3'), GAPDH (F: 5'-AGAAGGCTGGGGCTCA TTTG-3', R: 5'-AGGGGCCATCCACAGTCTTC-3'). We used primer dilution to determine primer efficiency. The data were normalized to the housekeeping gene GAPDH and calculated as 2^-ΔΔCT^.

### Immunohistochemistry (IHC)

Paraffin-embedded colon cancer tissues were cut into 3 μm sections for IHC. Immunohistochemistry kits were purchased from Beyotime (China). The slides were incubated overnight at 4°C with CK1ε and CK1δ antibody (Cell Signaling Technology USA), followed by incubation with secondary antibodies (Abm, China). The slides were incubated with DAB (3, 3-diaminobenzidine) and counterstained with Hematoxylin. Two researchers judged the results based on staining intensity.

### Cell culture

The human colon cancer cell lines RKO, LOVO, HCT116 and SW480 were obtained from the American Type Culture Collection (ATCC, USA). Cells were cultured in RPMI-1640 (Invitrogen, USA) with 10% FBS (fetal bovine serum, Gibco) at 37°C with a 5% CO_2_ atmosphere.

### MTT assay

Colon cancer cells were treatment by IC261 (HY-12774, MedChemExpress, USA) alone or combination with 2-DG ( HY-13966, MedChemExpress, USA). For cell viability detection, colon cancer cells were plated in 96-well plates at a density of 1.2×10^4^ cells/well in 200 μl complete medium. Cells were exposed to IC261 alone or combination with 2-DG for 24h. For cell proliferation detection, colon cancer cells were plated in 96-well plates at a density of 3×10^3^ cells/well in 200 μl complete medium. Cells were exposed to IC261 alone or combination with 2-DG and detected at 0h, 24h, 48h, 72h. Each treatment was repeated in six separate wells (n=6), 10 μl Cell Counting Kit-8 (CCK-8) assay (Dojindo, Japan) was added to each well and the plates were incubated additional for 2 h at the end of the experiments. Optical Density (OD) at a wave length of 450 nm was measured.

### Cell apoptosis assay

Colon cancer cells were plated in 6-well plates, the cells were incubated at 37 ℃with 5% CO_2_, Cells were added with different concentrations of inhibitors for apoptosis induction, cell apoptosis was evaluated using an Annexin V-FITC staining method according to the manufacturer's instructions (Dojindo, Japan). After cells were cultured with 20h, the culture medium was collected, and cells were washed with phosphate buffered solution (PBS) 3 times. Subsequently, cells in each group were incubated with 200 μL Annexin V-FITC binding buffer, 5 μL Annexin V-FITC, and 5μL propidium iodide (PI) with agitation. Then, the cells were stained for 20 minutes at room temperature in a dark room. Finally, quantifcation analysis of apoptotic cells was performed by flow cytometry (CytoFLEX, USA).

### Western blotting assay

Colon cancer cells were added with IC261 alone or combination with 2-DG. After cells were cultured with 20h, the total protein was extracted. And then protein concentrations were determined using the BCA protein assay (Beyotime, China). For western blotting analysis, equivalent amounts of protein (30 μg) were separated by 10% (w/v) SDS-polyacrylamide gel electrophoresis and transferred onto PVDF membranes (Bio-Rad, CA). The membranes were blocked with 5% (w/v) skim milk in buffer for 2h at room temperature and then incubated with primary antibodies (cleaved caspase-3, p53, TIGAR, G6PD were purchased from Cell Signaling Technology USA; Tublin was purchased from Sigma USA) overnight at 4℃, followed by incubation with secondary antibodies (Abm, China) at room temperature for 1h. Data were collected using a chemiluminescence imaging system (vilber, France) and image J softwire for quantitative analysis.

### Glucose metabolism RT2 profiler PCR array

Human Glucose Metabolism RT2 Profiler™ PCR Array kit was purchased from QIAGE (Germany) and operated following the instructions. Breifly, The total RNAs were extracted with QIAGE reagent according to the manufacturer's protocol. Then, the cDNAs were synthesized through reverse transcription (RT). The kit contains 84 key genes in glucose metabolism and determined by quantitative PCR using the Real Time PCR System (ABI7500, USA). The relatived expression of the 84 genes was presented using heat imaging with normalization to β-actin.

### Measurement of glucose uptake and lactate secretion

After treatment, the cell culture medium was collected. Glucose uptake was detected Glucose Assay Kit (Rsbio, China) according to the manufacturer's protocol. Briefly, glucose uptake is equal to the total amount of glucose in the medium minus the final glucose content in the cell culture medium. Lactate production was detected by Lactate Production Detection kit (KeyGen, China) according to the manufacturer's instructions. The results were counted by analyzing the difference of absorbance values (530nm).

### RNA interference and transient transfection

The siRNA against p53 were designed. The double-stranded sequences were: 5′AAGACUCCAGUGGUAAUCUAC-dTdT3′ (sense) and 5′GUAGAUUACCACUGGAGUCUU-dTdT3′ (anti-sense). Transfections were carried out by lipofectamine 3000 reagent (Invitrogen, USA). After 36h, colon cells were used for experiment.

### Statistics

SPSS 18.0 was used for statistical analysis. One-way analysis of variance (ANOVA) was used for three or more groups followed by t-tests. Rate comparison using chi-square test. Overall survival was estimated using the Kaplan-Meier method. Comparisons were considered to be statistically significant difference when *P* <0.05.

## Results

### The upregulation of CK1ε and CK1δ were correlated with advanced progression and poorer prognosis of colon cancer

**Figure 1:**
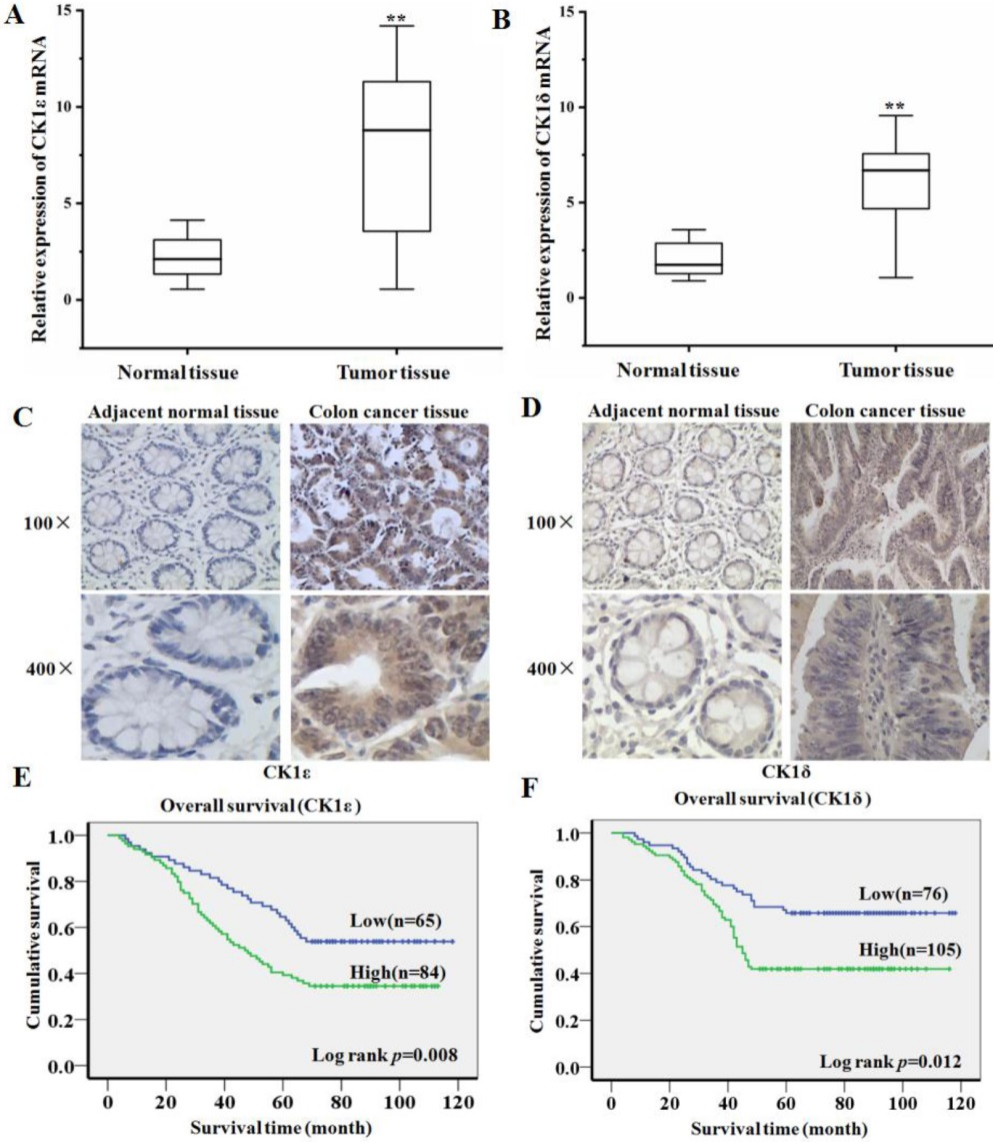
** CK1ε and CK1δ are upregulated in colon cancer and associated with poor clinical outcome. (A, B)** CK1ε and CK1δ mRNA were detected in colon cancer and paired adjacent normal tissues by using qRT-PCR. **(C, D)** CK1ε and CK1δ proteins were detected in colon cancer and paired adjacent normal tissues by using IHC. **(E)** Kaplan-Meier analysis of the CK1ε and CK1δ expression on overall survival of colon cancer patients (**p<0.01).

In order to explore the expression of CK1ε and CK1δ in colon cancer and its clinical significance, we first detected CK1ε and CK1δ mRNA expression in 45 colon cacner and paired adjacent normal tissues using qRT-PCR. Compared with normal tissues, the expression level of CK1ε and CK1δ in colon cancer tissues increased significantly (Figure 1A and 1B). The expression of CK1ε and CK1δ protein in colon cancer tissues was further detected by IHC. The result showed that CK1ε and CK1δ were mainly expressed in the cytoplasm and the expression of CK1 protein was upregulated in cancer tissues (Figure 1C and 1D). Analysis of protein results indicated that CK1ε and CK1δ protein expression level was correlated with TNM classification and differentiation (Table 1 and 2). Kaplan-Meier survival analysis showed that the patients with high CK1ε and CK1δ expression had significantly poorer overall survival than the patients with low CK1ε and CK1δ (Figure 1E and 1F). Together, these results suggested that CK1ε and CK1δ are highly expressed in colon cancer, which is significantly associated with advanced progression and poor prognosis.

### IC261 inhibited the survival and proliferation of colon cancer cells and induced apoptosis

**Figure 2:**
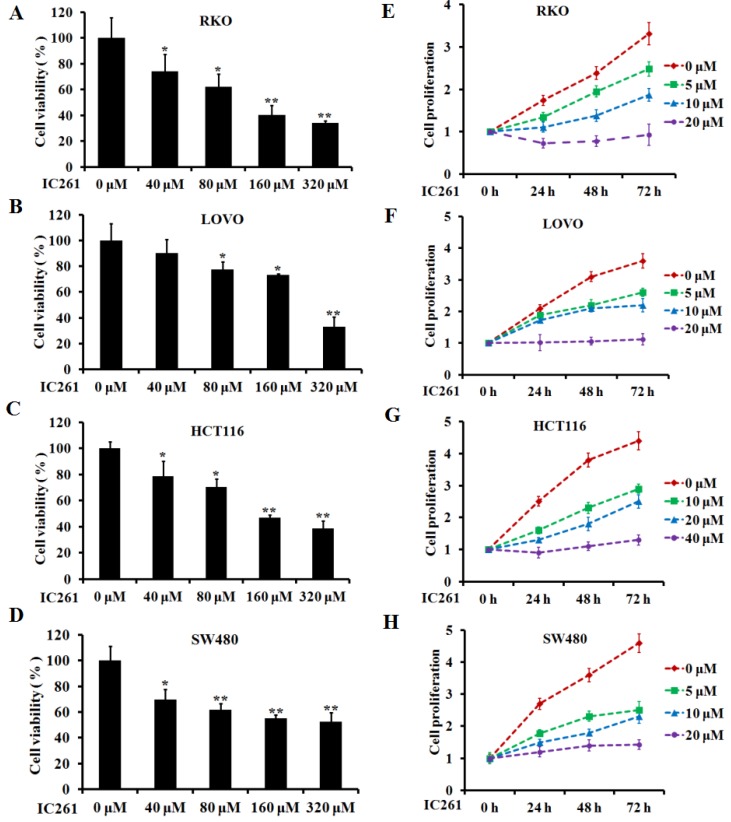
** IC261 inhibited the survival and proliferation of colon cancer cells. (A-D)** Cell viability of RKO, LOVO, HCT116 and SW480 was detected by MTT assay. **(C, D)** Cell proliferation of RKO, LOVO, HCT116 and SW480 was detected by MTT assay (*p<0.05, **p<0.01 vs 0 nM group).

**Figure 3:**
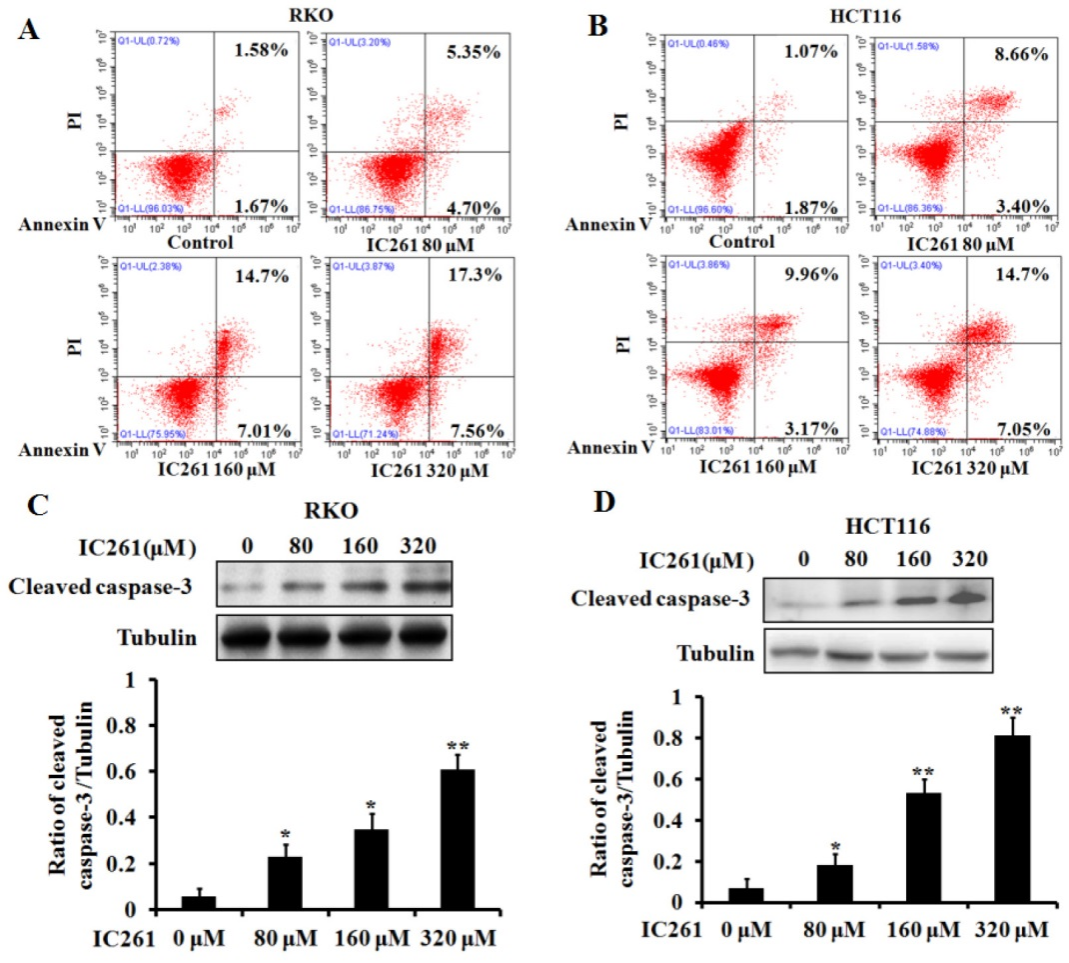
** IC261 induced apoptosis of colon cancer cells. (A, B)** Apoptotic rate of RKO and HCT116 induced by IC261 were detected by flow cytometry. **(C, D)** The expression of cleaved caspase-3 of RKO and HCT116 induced by IC261 was detected by using Western blotting (*p<0.05, **p<0.01 vs 0 nM group).

Based on the above results, we used CK1ε/δ-specific inhibitor IC261 to investigate the effects of CK1ε and CK1δ on survival, proliferation and apoptosis of colon cancer cells. Four colon cancer cell lines (RKO, LOVO, HCT116 and SW480) were selected and given IC261 at different concentrations. The results showed that IC261 could significantly reduce the cell viability and proliferation (Figure 2). We further examined the effect of IC261 on apoptosis of colon cancer cells. The results showed that IC261 could significantly increase the apoptotic rate and cleaved caspase-3 expression (Figure 3). To sum up, these data revealed that IC261 can induce obvious cytotoxicity of colon cancer cells.

### IC261 regulated aerobic glycolysis of colon cancer cells

**Figure 4:**
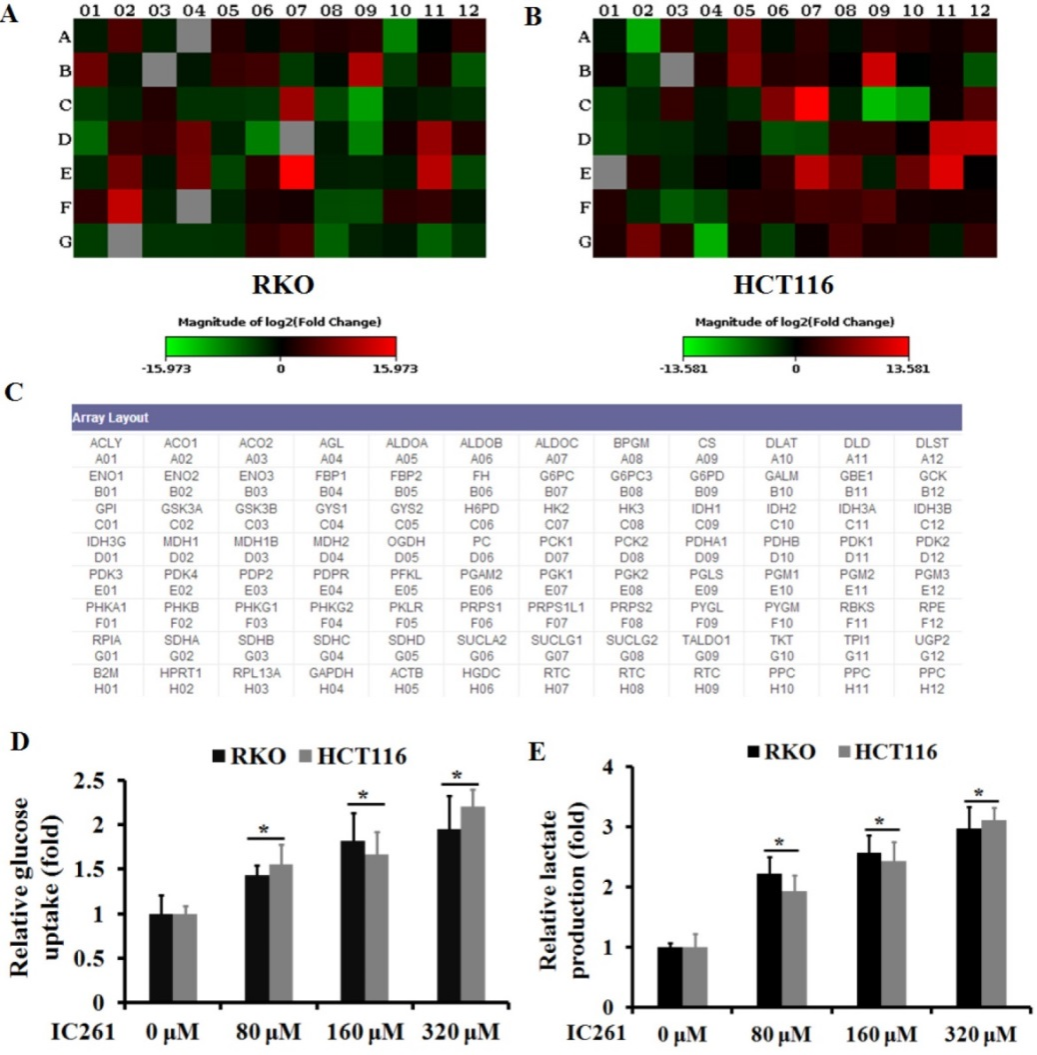
** IC261 increased the level of aerobic glycolysis in colon cancer cells. (A, B)** Glucose metabolism in RKO and HCT116 treatment with IC261 was examined by PCR array. **(C)** The list of glucose metabolism gene was provided by RT2 Human Glucose Metabolism Profiler PCR array. **(D)** Glucose uptake was measured in RKO and HCT116 treated with IC261. **(E)** Lactate production was measured in RKO and HCT116 treated with IC261 (*p<0.05 vs 0 nM group).

Unlike normal cells, cancer cells gain energy preferentially through aerobic glycolysis, known as the Warburg effect. We then investigated whether IC261 can affect aerobic glycolysis in colon cancer cells. The RT2 Human Glucose Metabolism Profiler PCR array was used to explore the effect of IC261 on colon cancer cells RKO and HCT116. The results showed that the glycolysis-related genes PGM2, PDK1, PGK1, HK2 and G6PD increased in both cell lines when IC261 was administrated,. On the contrary, the expression level of TCA cycle related IDH1, DLAT and PDHA1 decreased (Figure 4A-C). Next, we detected the glucose uptake and the lactate production in RKO and HCT116 cells treated by IC261. As shown in Fig. 4D and E, IC261 can significantly increase cell glucose uptake and lactate production. These results suggest that IC261 can increase the level of aerobic glycolysis in colon cancer cells.

### IC261 modulates aerobic glycolysis in colon cancer cells through p53-dependent mechanism

**Figure 5:**
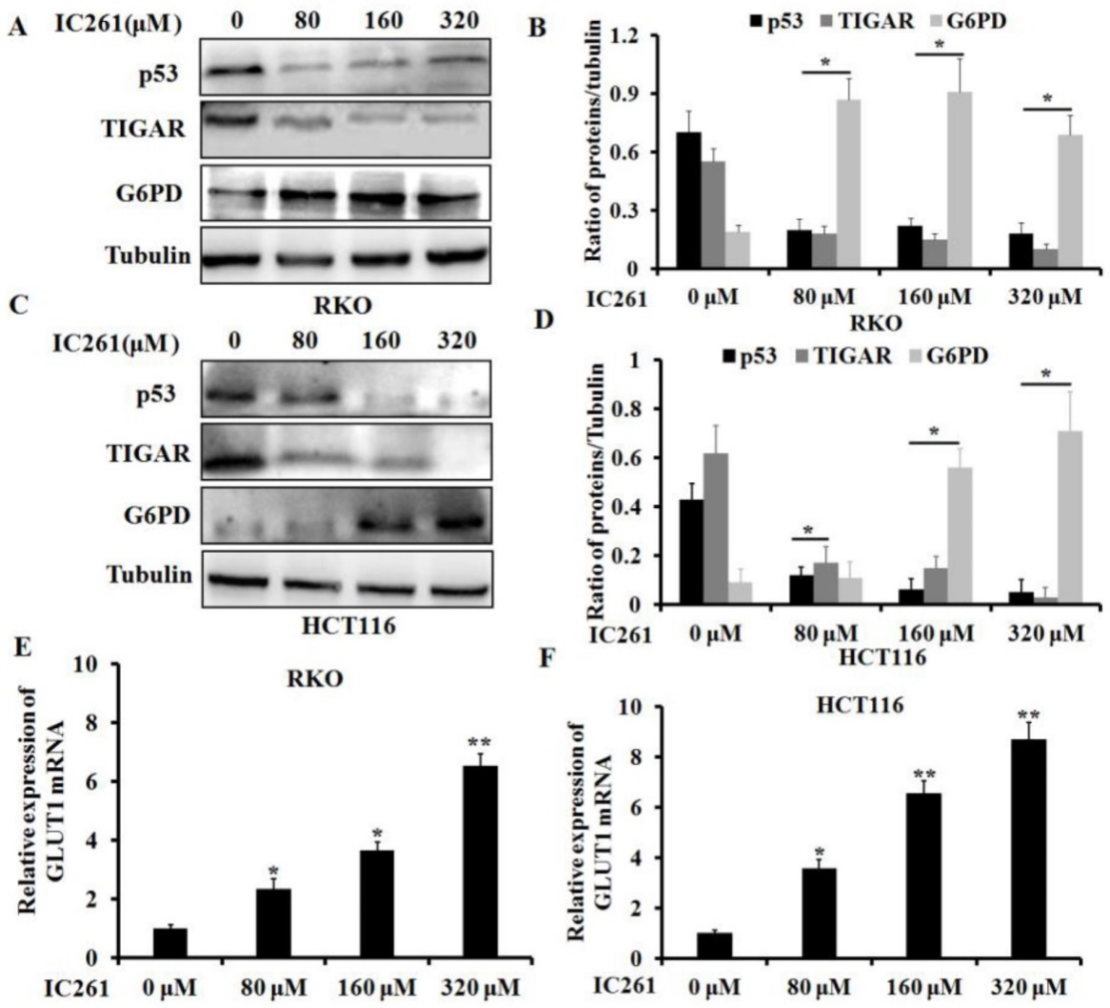
** IC261 regulated the glycolysis related proteins and gene of colon cancer cells. (A, C)** The expression of p53, TIGAR and G6PD of RKO and HCT116 induced by IC261 was detected by using Western blotting. **(B, D)** Quantitive analysis of A and C. **(E, F)** GLUT1 mRNA expression in colon cancer treated with IC261 was detected by using qRT-PCR (*p<0.05, **p<0.01 vs 0 nM group).

**Figure 6:**
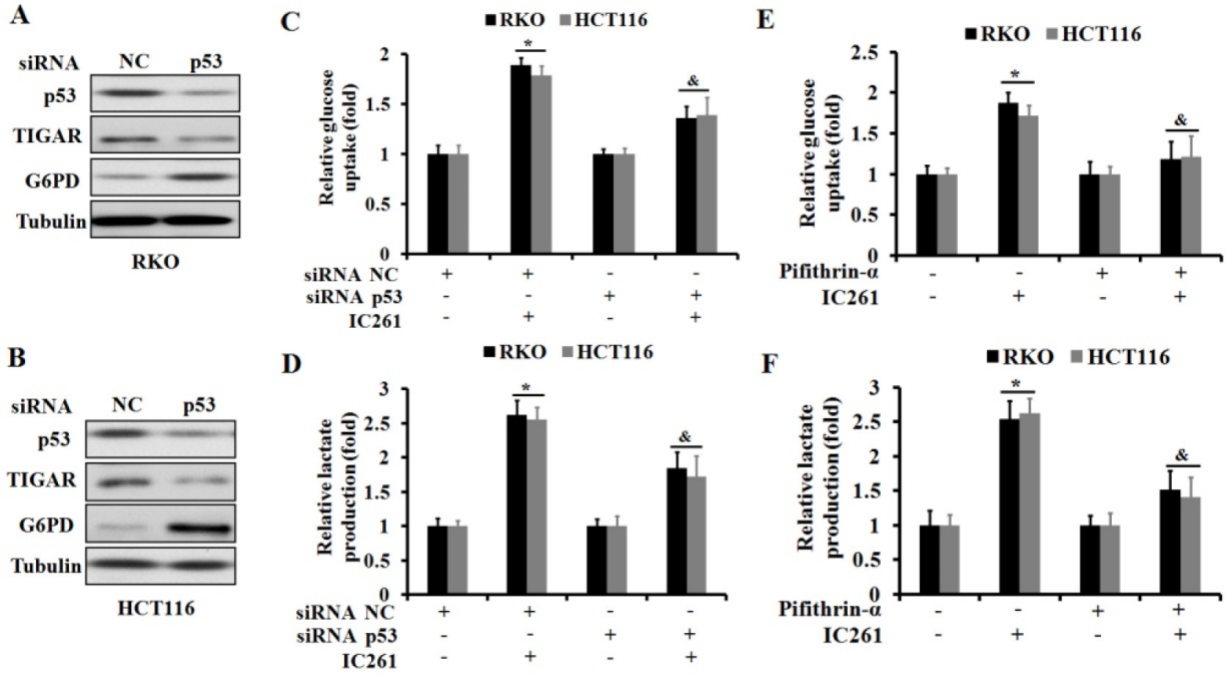
** IC261 modulates aerobic glycolysis in colon cancer cells through p53-dependent mechanism. (A, B)** The expression of p53, TIGAR and G6PD of RKO and HCT116 in NC and p53 siRNA group was detected by using Western blotting. **(C)** Glucose uptake was measured in RKO and HCT116 treated by IC261 with NC or p53 siRNA. **(D)** Lactate production was measured in RKO and HCT116 treated by IC261 with NC or p53 siRNA. **(E)** Glucose uptake was measured in RKO and HCT116 treated by IC261 with or without p53 inhibitor Pifithrin-α. **(F)** Lactate production was measured in RKO and HCT116 treated by IC261 with or without p53 inhibitor Pifithrin-α (*p<0.05 vs control group, ^&^*p*<0.05 vs IC261 group).

As a transcription factor, p53 has been shown to be involved in regulating cancer cell metabolism in recent years 23. We further clarified whether IC261 regulates aerobic glycolysis in colon cancer cells through p53. We selected three genes TIGAR (TP53-induced glycolysis and apoptosis regulator) 24, G6PD (glucose-6-phosphate dehydrogenase) 25 and GLUT1 (glucose transporter 1) 26, which are closely related to the regulation of cancer cell metabolism by p53. The results showed that IC261 could significantly reduce the expression of p53 and TIGAR proteins and increase the expression of G6PD protein in RKO and HCT116 cells (Figure 5A-D).As shown in Fig. 5E and F, IC261 could significantly the expression of GLUT1 mRNA. To further clarify the role of p53 in the regulation of aerobic glycolysis in colon cancer cells by IC261, we constructed p53 siRNA. The results showed that inhibiting p53 could significantly reduce the expression of TIGAR and increase the expression of G6PD (Figure 6A and 6B). The combination of p53 siRNA could significantly alleviate the increase of glucose uptake and lactate production in colon cancer cells induced by IC261 (Figure 6C and 6D). In addition, we used p53 inhibitors Pifithrin-α. The results showed that Pifithrin-α could significantly reduce the increase of aerobic glycolysis induced by IC261 in colon cancer cells (Figure 6E and 6F). These results suggested that IC261 may affect the aerobic glycolysis level of colon cancer cells by regulating P53 expression.

### Inhibition of aerobic glycolysis can significantly increase the cytotoxicity induced by IC261 in colon cancer cells

**Figure 7:**
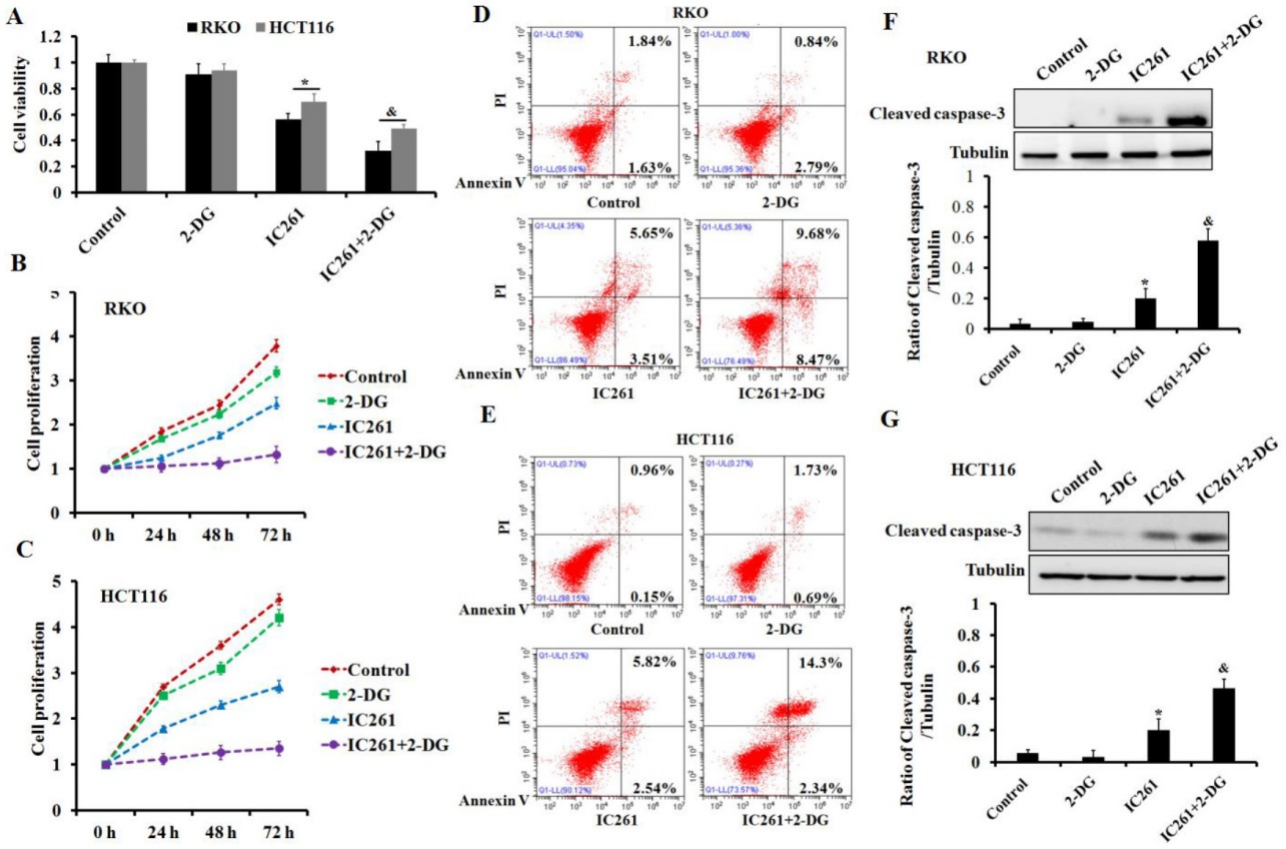
** Inhibition of aerobic glycolysis increased the cytotoxicity induced by IC261 in colon cancer cells. (A)** The cell viability was measured in RKO and HCT116 treated by IC261(80 μM) with or without aerobic glycolysis inhibitor 2-DG (2 mM). **(B, C)** The cell proliferation was measured in RKO and HCT116 treated by IC261 with or without 2-DG. **(D, E)** Apoptotic rate of RKO and HCT116 treated by IC261 with or without 2-DG was detected by using flow cytometry. **(F, G)** The expression of cleaved caspase-3 of RKO and HCT116 treated by IC261 with or without 2-DG was detected by using Western blotting (*p<0.05 vs control group, ^&^*p*<0.05 vs IC261 group).

To clarify the role of aerobic glycolysis in IC261-induced cytotoxicity of colon cancer, glycolysis inhibitor 2-DG was used. The results showed that 2-DG could further reduce the cell survival and proliferation inhibition induced by IC261 (Figure 7A-C). Further results showed that the combination of 2-DG could aggravate the apoptosis of colon cancer cells induced by IC261 (Figure 7D-G). These results indicated that cell aerobic glycolysis may play a protective role in IC261-induced cytotoxicity.

## Discussion

CK1ε and CK1δ, showed the highest homology, are closely related isoforms of CK1 which is highly conserved in the kinase domain 10. It is reported that they play an important role in the regulation of various cellular biological behaviors, especially in cancer 9, 27. Studies have shown that CK1ε can affect the proliferation of breast cancer cells by regulating the mRNA translation process 28. CK1δ participates in the degradation of Brg1 protein in gastric cancer cells and regulates metastasis 29. Our results showed that the expression of CK1ε and CK1δ were significantly increased either at the level of mRNA or protein in colon cancer tissues. The upregulation of CK1ε and CK1δ were closely correlated with the poor differentiation, advanced TNM stage and poor prognosis of colon cancer. These results suggest that CK1ε and CK1δ may be involved in the development of colon cancer, and targeting CK1ε and CK1δ may affect the biological behavior of colon cancer cells.

IC261 is a selective ATP-competitive inhibitor of CK1ε/δ with less effect on CK1a 30. It has been reported that IC261 is involved in the regulation of tumor cell survival and proliferation 14, 31-33. However, the effect of IC261 on the biological behavior of colon cancer cells remains unknown. Four colon cancer cell lines were selected and treated with IC261. The results showed that IC261 could significantly reduce the survival and proliferation of colon cancer cells. Meanwhile IC261 can increase the apoptotic level of colon cancer cells.

In recent years, more and more researches demonstrated that aerobic glycolysis plays a pivotal role in the development of colon cancer and in the treatment of colon cancer by using anticancer agent 34-36. However, it is not clear whether IC261 are involved in the regulation of aerobic glycolysis in colon cancer cells. Our investigations showed that IC261 increased glycolysis-related genes and decreased the TCA cycle related genes. In addition, IC261 can significantly increase cell glucose uptake and lactate production, suggesting that the level of aerobic glycolysis increased. Although we have revealed the role of IC261 in the aerobic glycolysis, the underlying mechanism is unknown.

CK1 family members play an important role in DNA damage regulation 37. Interestingly, P53 activation ensures genomic stability 38,39. This signaling network makes CK1 associated with p53 40. It has been reported that CK1ε and CK1δ, including CK1a, can phosphorylate p53 at different sites (Ser-6, Ser-9, Ser-15 and Ser-20) 41-44. The modification of p53 by CK1ε and CK1δ can reduce its binding with MDM2 (Mouse double-minute 2 homolog) and activate p53 45. In addition, CK1δ can phosphorylate MDM2, reduce its interaction with p53, and further stabilize and activate p53 46.However, the effect of CK1ε/δ inhibitors IC261 on the expression of p53 in colon cancer cells remains unclear.Our results show that IC261 can significantly reduce the level of p53 protein in colon cancer cells.

As a transcription factor and tumor-suppressor, p53 participates in the regulation of cellular biological behavior by targeting a variety of genes, including cell growth, cycle and apoptosis 47. Recent studies have shown that p53 can also participate in the regulation of tumor cell metabolism, especially glucose metabolism 23. P53 is involved in the regulation of aerobic glycolysis in cancer cells by regulating various metabolic related genes. It was reported that p53 inhibited glycolysis by regulating TIGAR (TP53-induced glycolysis and apoptosis regulator) expression 24. p53 inhibits glycolytic intermediates into the PPP (pentose phosphate pathway) by binding to G6PD (glucose-6-phosphate dehydrogenase) and inhibiting its activity 48. In addition, p53 reduces glucose uptake by inhibiting GLUT1 and GLUT4 (glucose transporter 4) levels at the transcriptional level 26. Our results showed that IC261 could decrease the protein expression of TIGAR and increase the protein expression of G6PD in colon cancer cells. Meanwhile, IC261 can increase the mRNA expression of GLUT1. To further clarify the role of p53 in the regulation of aerobic glycolysis in colon cancer cells by IC261, we used p53 siRNA and inhibitors, and found that inhibition of p53 could reduce the aerobic glycolysis induced by IC261. To investigate the role of aerobic glycolysis in IC261-induced colon cancer cell death and apoptosis, we combined IC261 and 2-DG, an inhibitor of aerobic glycolysis. The results showed that combined application could further increase the cytotoxicity induced by IC261, suggesting that aerobic glycolysis played a protective role in IC261-induced cell death.

In conclusion, we demonstrated that CK1ε and CK1δ are highly expressed in colon cancer tissues and are closely related to clinical significance. CK1δ/ε inhibitors IC261 increased of the cytotoxicity and aerobic glycolysis of colon cancer cells. IC261 induced aerobic glycolysis in colon cancer cells is p53-dependent pathway. Inhibition of aerobic glycolysis can further increase the cytotoxicity induced by IC261. The study may provide a new scheme and combined drug strategy for clinical treatment of colon cancer.

## Figures and Tables

**Table 1:** Correlation between the clinicopathological features and CK1ε expression

Characteristics	N	CK1ε expression		*P*
		Low	High	
**Gender**				0.322
Male	72	28	44	
Female	77	37	40	
**Age**				0.566
≥55	94	41	53	
< 55	55	24	31	
**T stage**				0.003**
T1-T2	68	39	29	
T3-T4	81	26	55	
**N stage**				0.012*
Nx-0	62	35	27	
N1-2	87	30	57	
**M stage**				0.001**
M0	68	40	28	
M1	81	25	56	
**Differentiation**				0.002**
Well	51	32	19	
Moderately	45	17	28	
Poorly	53	16	37	

**Table 2:** Correlation between the clinicopathological features and CK1δ expression

Characteristics	N	CK1δ expression		*P*
		Low	High	
**Gender**				0.65
Male	89	39	50	
Female	91	36	55	
**Age**				0.642
≥55	112	45	67	
< 55	68	30	38	
**T stage**				0.015*
T1-T2	81	42	39	
T3-T4	99	33	66	
**N stage**				0.007**
Nx-0	86	45	41	
N1-2	94	30	64	
**M stage**				0.015*
M0	90	46	44	
M1	90	29	61	
**Differentiation**				0.001**
Well	56	35	21	
Moderately	59	20	39	
Poorly	65	20	45	
